# New Research on MEMS Acoustic Vector Sensors Used in Pipeline Ground Markers

**DOI:** 10.3390/s150100274

**Published:** 2014-12-25

**Authors:** Xiaopeng Song, Zeming Jian, Guojun Zhang, Mengran Liu, Nan Guo, Wendong Zhang

**Affiliations:** 1 Key Laboratory of Instrumentation Science & Dynamic Measurement, Ministry of Education, North University of China, Taiyuan 030051, China; E-Mails: sroc@163.com (X.S.); guonan0902@163.com (N.G.); 2 Science and Technology on Electronic Test & Measurement Laboratory, North University of China, Taiyuan 030051, China; E-Mail:liumengran1991@163.com (M.L.)

**Keywords:** MEMS acoustic vector sensor, pipeline inspection gauge, above-ground marker, positioning

## Abstract

According to the demands of current pipeline detection systems, the above-ground marker (AGM) system based on sound detection principle has been a major development trend in pipeline technology. A novel MEMS acoustic vector sensor for AGM systems which has advantages of high sensitivity, high signal-to-noise ratio (SNR), and good low frequency performance has been put forward. Firstly, it is presented that the frequency of the detected sound signal is concentrated in a lower frequency range, and the sound attenuation is relatively low in soil. Secondly, the MEMS acoustic vector sensor structure and basic principles are introduced. Finally, experimental tests are conducted and the results show that in the range of 0°∼90°, when *r* = 5 m, the proposed MEMS acoustic vector sensor can effectively detect sound signals in soil. The measurement errors of all angles are less than 5°.

## 1. Introduction

Pipeline transport has become the leading mode of transportation of oil resources because of its unique advantages of stability, safety, uninterrupted use and low economic cost [1], but as pipelines age, along with construction defects, artificial damage and corrosion, pipeline leakage accidents occur frequently [2,3]. This is a serious threat to the environment and human security, therefore, pipeline fault detection is particularly crucial.

Currently, the most widely used and effective detection method is to record pipe defects by pipeline inspection gauges (PIGs) [4]. However, the errors caused by its odometer wheel and the changes in diameter resulting from unavoidable wear and other factors, inevitably reduce the positioning accuracy [4], so it's difficult to repair the pipeline precisely and a timely way. In order to solve this problem, the PIG AGM technology comes into being. With the widespread use of PIGs, various research institutions and manufacturers have paid more attention to the study of pipe AGM systems. So far, various principles such as the eddy current method, magnetic and sound methods are used in developing AGM techniques [5].

The AGM system based on eddy current effect was first applied in pipeline detection technology. Its disadvantages such as small detection range, shallow depth and being heavily affected by the coil position, have seriously restricted the widespread popularization of this method [6]. In the recent three decades, AGM systems based on the Hall magnetic sensor detection principle have also been put forward. Compared with eddy current-type AGM systems, they provide increased detection depth and decreased power consumption [7], hence, they have been widely used. With the development of pipeline technology, the thickening of pipeline walls and the increased burial depths make it difficult for magnetic induction lines to penetrate through the pipeline walls, thus the signals received from sensors are weaker, making it difficult to realize precision PIG measurements with this method. In recent years, in the US acoustic sensors have been used to realize PIG AGM by detecting the friction signal between the pipeline wall and the PIG, without any consideration of the electromagnetic principle. AGM systems based on the acoustic principle have large induction scope and high accuracy [8].

Using sound methods has become the consensus of many institutions and the major trend of technological development [8,9]. Currently, piezoelectric ceramic vibration sensors or moving-coil geophones are usually used in the PIG AGM [10]. Because of the traditional sensors' poor consistency, low sensitivity, the fact they are easily affected by the soil environment and their weak anti-jamming ability, better sensors are needed to realize precise PIG positioning. Hence, a novel MEMS acoustic vector sensor applied in AGM systems is proposed in this paper. The MEMS vector acoustic sensor, compared with the other two traditional kinds of sensors, has higher sensitivity, signal-to-noise ratio and resolution [10]. It has great feasibility to receive weak signals in soil environments [10] and further, the good orientation ability of MEMS acoustic vector sensors is verified in soil in this paper.

## 2. AGM Principle Based on Sound Signals

**Figure 1.**
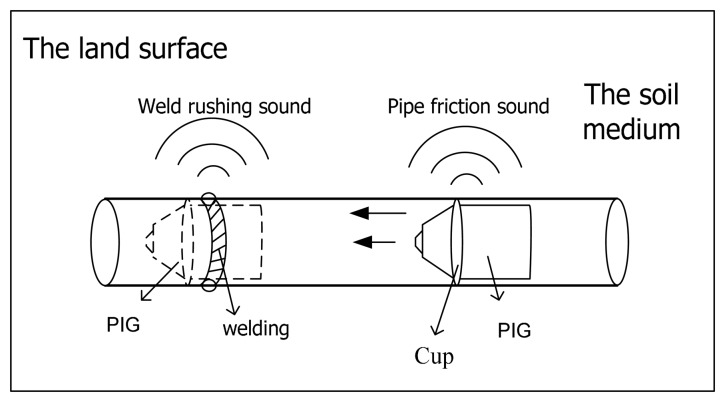
Sound generating principle of a PIG in a pipe.

When PIGs run in pipes, errors of about 1 m per 1 km will be produced. The longer the pipe, the greater the cumulative error, and the defect positioning is less accurate, thus requiring more repair work, manpower and material resources in the future [11], so for long distance pipeline defect detection, it is necessary to mark the PIG every kilometer. When the PIG works in the pipe, it will produce two kinds of acoustic signal: one kind is a friction sound signal because of friction between the PIG and pipe, the other is the crash sound signals induced by the PIG and welding (as shown in Figure 1). In general, the friction sound signal frequency is concentrated in the 150 Hz∼380 Hz range, and crash sound signal frequency is concentrated in the dozens of Hertz range [11].

When the PIG runs in the pipe, sound signals will be transmitted all around by the soil medium. Soundwave energy attenuates exponentially with distance when soundwaves propagate into the soil. The higher the frequency of the sound wave is, the greater the attenuation is [12,13]. Due to the soil attenuation effect, the signal will be very weak. The selection of appropriate sensors to collect these weak acoustic signals due to soil attenuation is becoming the biggest problem of AGM.

## 3. MEMS Acoustic Vector Sensor

**Figure 2.**
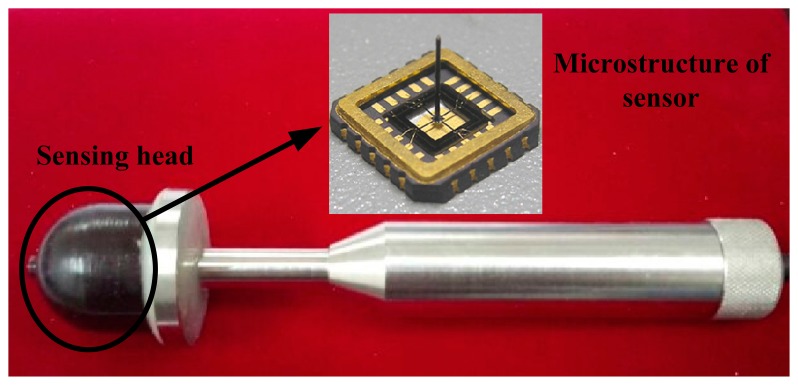
MEMS acoustic vector sensor.

A MEMS acoustic vector sensor based on the piezoresistive principle can measure low frequency down to zero. High sensitivity, good performance at low frequency and low power consumption make it a unique technology for measuring weak signals [14–17]. The MEMS vector sensor is shown in Figure 2.

**Figure 3.**
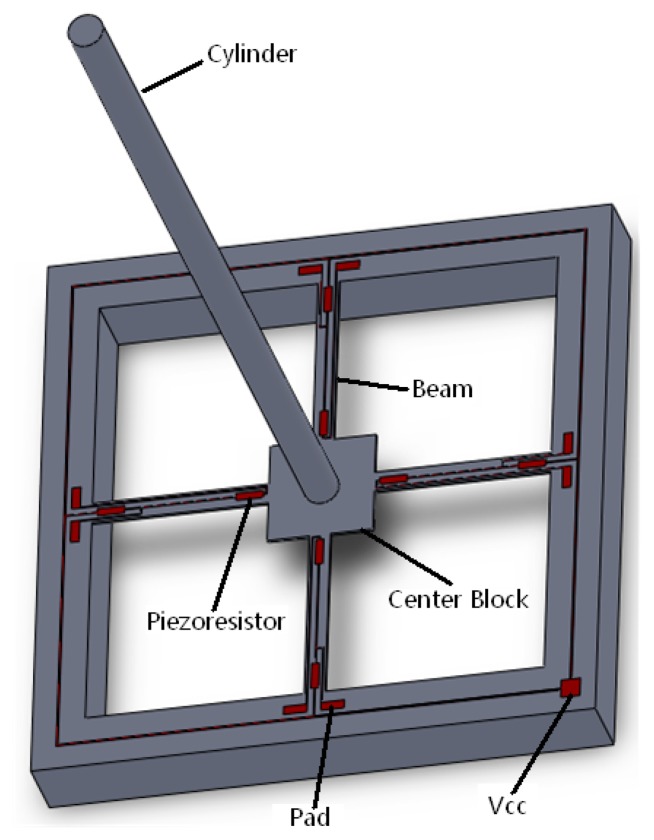
Microstructure model graph of sensor.

The microstructure of a MEMS acoustic vector sensor is based on silicon and composed of a four-arm silicon microstructure made by a standard piezoresistive silicon micromachining process and a rigid cylindrical body fixed in the center of the beam. The four-arm silicon microstructure can be seen as four cantilevers. A structure model is shown in Figure 3.

**Figure 4.**
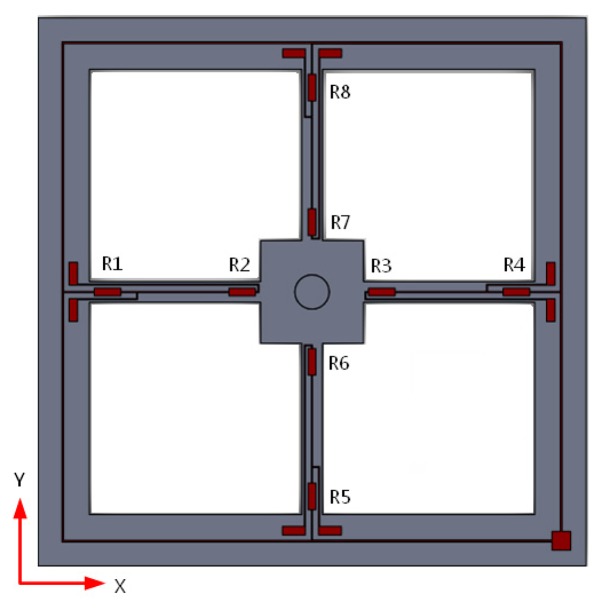
Diagram of the distribution of the piezoresistor.

**Figure 5.**
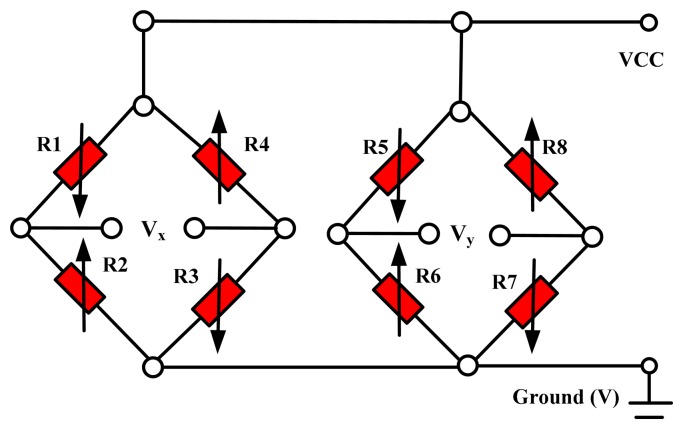
Scheme of the Wheatstone bridge.

There are eight equal-value strain varistors, R1, R2, R3, R4, R5, R6, R7 and R8, made by means of diffusion in the four-arm system. R1, R2, R3 and R4 constitute a Wheatstone bridge, and R5, R6, R7 and R8 constitute another. The distribution of piezoresistors on the micro-structure is shown in Figure 4 and the Wheatstone bridge is shown in Figure 5. When an arbitrary signal acts on the sensor, it can be decomposed into X-direction and Y-direction components, deforming the cantilevers and the Wheatstone bridges are changed. According to the outputs (V_x_ and V_y_) of the Wheatstone bridge changes, we can determine the direction of the signal source.

The MEMS acoustic vector sensor sensitivity is −170 dB and the frequency band is 0∼1000 Hz [18–20]. The sound signal frequency range which is researched in the experiments is mainly concentrated in the dozens of Hertz to 380 Hz range, so the sensor can effectively detect the signals in that frequency range.

## 4. Directional Principle of MEMS Acoustic Vector Sensor

### 4.1. Directional Basic Principles

**Figure 6.**
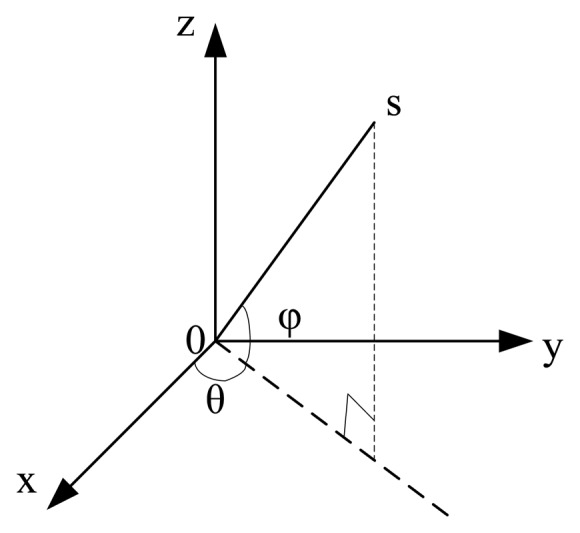
MEMS acoustic vector directional model.

The directional measurement module of the MEMS acoustic vector sensor is shown in Figure 6, where “s” is position of the sound source, “φ” is the pitch angle between sound signal and horizontal plane, and “θ” is horizontal angle between the projection of “s” and x-axis. In an acoustic vector field, each point can be decomposed into four components: medium particle vibration velocity components *v_x_*(*r*,*t*), *v_y_*(*r*,*t*), *v_z_*(*r*,*t*) and sound pressure road *p*(*r*,*t*). The acoustic vector sensor can get the above four components by synchronously, concurrently and independently measuring the acoustic field. We use *v*(*r*,*t*) to depict the particle vibration velocity, then from Figure 6, we can get the following formulas:

(1) $$\{\begin{array}{l} p\left(r,t\right)=v\left(r,t\right)\\ v_{x}\left(r,t\right)=v\left(r,t\right)\cos\theta \sin\varphi \\ v_{y}\left(r,t\right)=v\left(r,t\right)\sin\theta \sin\varphi \\ v_{z}\left(r,t\right)=v\left(r,t\right)\cos\varphi \end{array}$$

In the above formulas: the range of “θ” is [0, 2π], and “φ” is [0, π]. From Equation (1), we can find the particle vibration velocity components *v_x_*, *v_y_*_._and *v_z_*, then we can separately work out the horizontal angle “θ” and the pitch angle “φ” from Equations (2) and (3):

(2) $$\theta =\arctan\left(\frac{v_{y}}{v_{x}}\right)$$

(3) $$\varphi =\text{arc}\tan\left(\frac{v_{z}}{\sqrt{v_{x}^{2}+v_{x}^{2}}}\right)$$

The measurement signal of the acoustic vector sensor is a voltage component, which is proportional to the vibration velocity components above. Therefore, it is easy to find its horizontal angle and pitch angle if we measure the voltage component. These are the basic directional principles of a MEMS acoustic vector sensor. When positioning the PIG, we just need to get the horizontal angle precisely, and not pitch angle calculations are not involved.

### 4.2. Beam-Forming Algorithm

**Figure 7.**
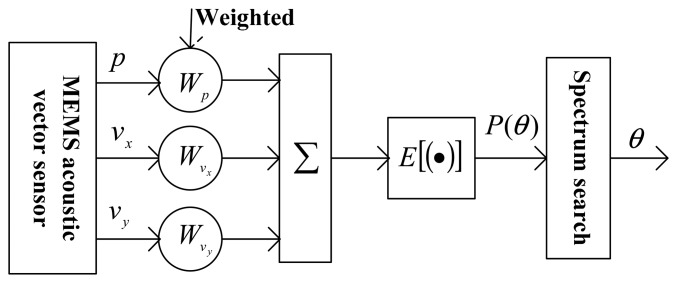
Beam-forming principle block diagram.

Direction of Arrival (DOA) estimation is one of the most critical steps in sound signal processing. For a single vector sensor, the beam-forming algorithm is chosen in this paper. The beam-forming algorithm processes (weigh, delay and sum) the output signals of sensor and then gives the spatial directionality. The maximum of the spectral peak is the DOA estimation result. The block diagram of the principle is shown in Figure 7.

In Figure 7, the three way signal *p* (*t*), *v_x_*(*t*) and *v_y_* (*t*) output of MEMS acoustic vector are weighted by 1, cosθ and sinθ, then summing them. The result is:

(4) $$y\left(t\right)=p\left(t\right)+v_{x}\left(t\right)\cos\theta +v_{y}\left(t\right)\sin\theta$$

The average power of *y*(t) is:

(5) $$P\left(\theta \right)=E\left[\left|y{\left(t\right)}^{2}\right|\right]$$

where, *E* []-ensemble average; *P*(θ)-space spectrum of the output, is a function of θ. Only when θ = θ_0_, P(θ) can acquire the max by searching the max of *P*(θ) in θ ∊ [0, 2π], thus θ_0_ is the azimuth angle of the sound signal [21,22].

## 5. Experiments

**Figure 8.**
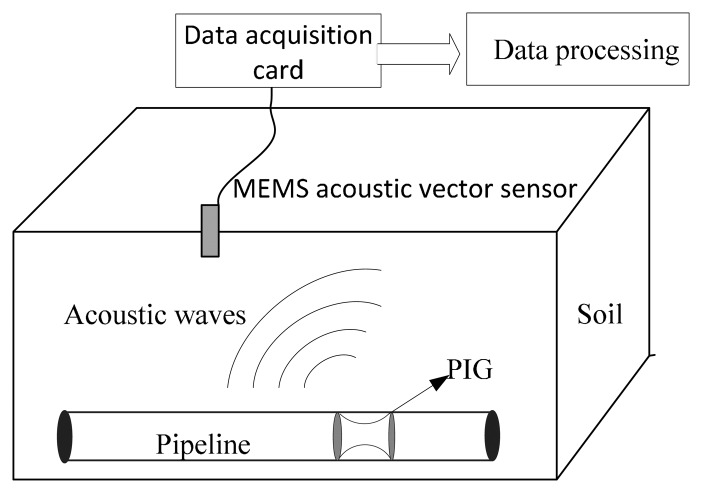
The positioning principle of the PIG based on a MEMS acoustic vector sensor.

In order to verify the orientation of the MEMS acoustic vector sensor, experiments were conducted in soil. The experimental site is located on flat, open land in Taiyuan, Shanxi Province, China, where the soil properties are as follows: porosity, 35%–40%; specific gravity, (2.6–2.7) × 10^3^ kg/m^3^; bulk density, 1.2–1.3; moisture content, 48%–55%. The positioning principle of the PIG based on the MEMS acoustic vector sensor is shown in Figure 8.

**Figure 9.**
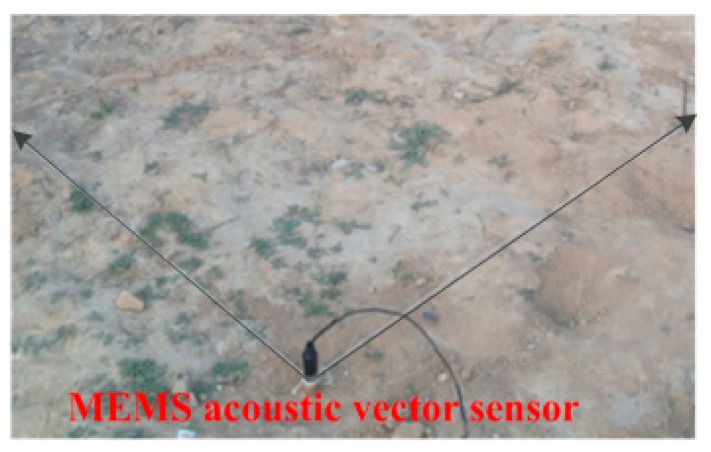
Sensor burial mode.

In the experiment, the sensor is vertically downward, and two thirds of the sensor (including sensing head) is buried tightly and heavily in the soil (as shown in Figure 9), ensuring that the sensor is well coupled with the soil. We use a steel hammer knocking on the ground to stimulate the sound source.

**Figure 10.**
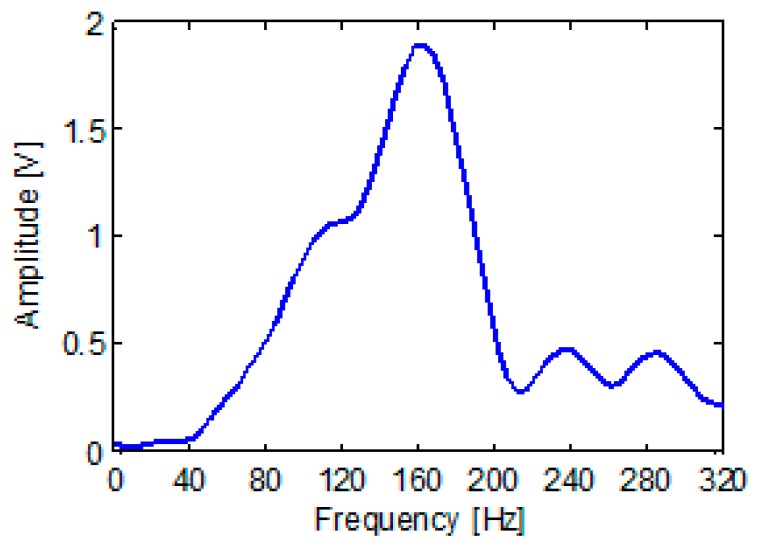
The spectral analysis of the knocking signal.

By collecting and analyzing the knocking signal with a NI data acquisition card (model: PXTe-1071, National Instruments, Austin, TX, USA) it can be found that the knocking signal is in 80 Hz–300 Hz range (Figure 10) which falls in the spectral range of the friction signal of the PIG running in the pipeline. To a certain extent, a knocking signal could simulate the actual sound signal.

**Figure 11.**
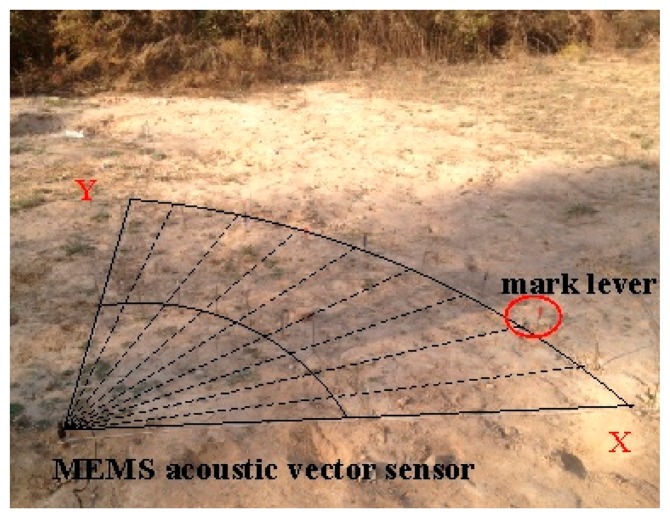
Experiment site.

During the experiment, taking the sound sensor as the center and r as the radius, we can get a semicircle, and dividing it on average by 10° as bisecting angle, each point represents a degree. The MEMS acoustic vector sensor has been buried in soil the as shown in Figure 11, with the X-direction coinciding with 0° and the Y-direction coinciding with 90°.

**Figure 12.**
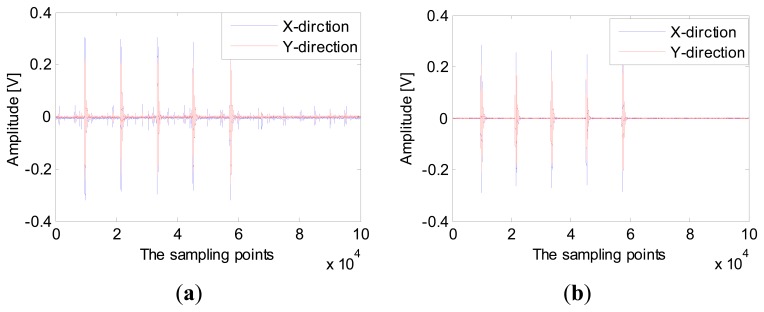
Signal collected by MEMS acoustic vector sensor (**a**) The original signal; (**b**) the filtered signal.

Knocking the ground in the equal diversion point, the sensor will translate the collected sound signal into an electrical signal. The electrical signals are collected by the data acquisition card and processed in MATLAB. The data acquisition card is a PXTe-1071 from NI, and the terminal box is a BNC2110. The experimental sampling rate is 10 K, and the signal collection time is 10 s. Knocking equal diversion points at 40° (*r* = 5m), the signal collected by sensor is shown in Figure 12a. The signal processed by the wavelet denoising method is shown in Figure 12b.

**Figure 13.**
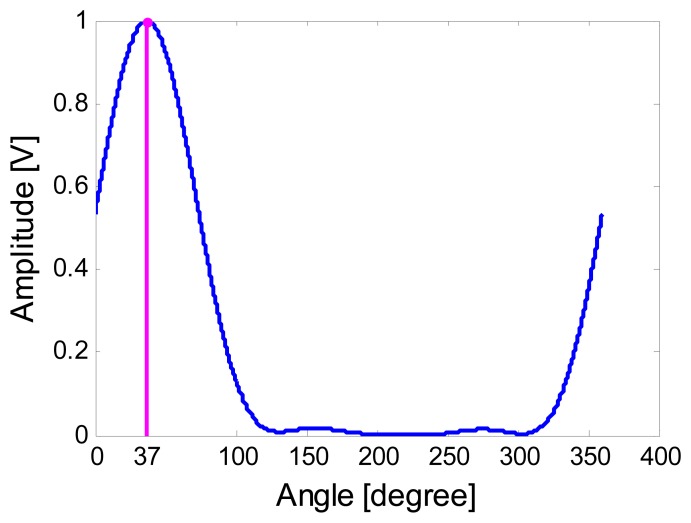
Azimuth estimation based on the acoustic vector sensor.

DOA estimation is one of the most critical steps in sound signal processing. For a single vector sensor, the beam-forming algorithm (as described above) is chosen in this paper. The DOA estimation result of the sound source was obtained by the beam-forming method when the actual azimuth angle is 40°. The experimental result is shown in Figure 13.

**Figure 14.**
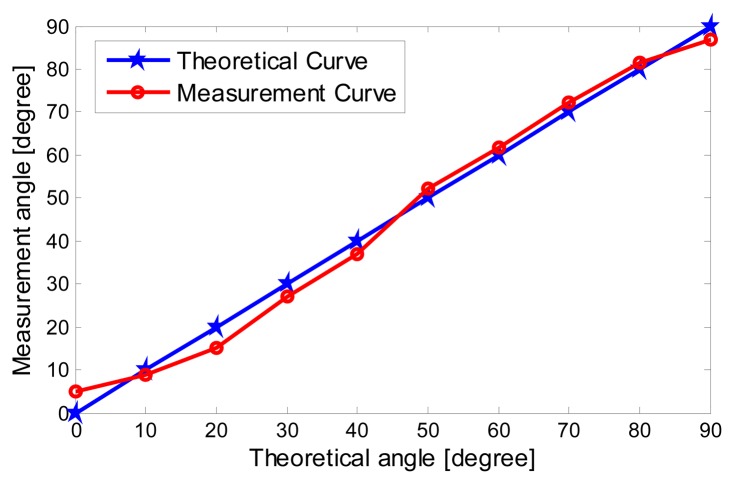
Correlation curve of practical angle and theoretical angle.

The experiment result in Figure 13 shows that when the actual azimuth angle of the sound source is 40°, the experimental result is 37° so the error is 3°. Further, in the range of 0°∼90°, *r* = 5 m, we knock five times on each point. Processing the experimental results, taking the average gives the estimation results shown in Table 1 The correlation curves of the measurement angle and theoretical angle are shown in Figure 14.

The experimental results show that in the range of 0°∼90°, the MEMS acoustic vector sensor can effectively detect the sound signals in soil. The measurement errors of all angles are less than 5°.

## 6. Conclusions

A MEMS acoustic vector sensor is innovatively applied in a pipeline detection system. It represents a bold and effective attempt to spanning from the water to the land. Through field experiments, in the range of 0°∼90°, when *r* = 5 m, the MEMS acoustic vector sensor can effectively detect the sound signals in soil, and the measurement errors of all angles are less than 5°. The application of the acoustic vector sensor used in pipeline detection system has been primarily verified. However, to make full use of its characteristics such as high sensitivity and good directivity, further research on the AGM of the PIG is needed so as to achieve more accurate orientation.

## Figures and Tables

**Table 1.** Measurement angle by MEMS acoustic vector sensor.

**Theory Angle (Degree)**	**Measurement Angle (Degree)**	**Error (Degree)**	**Standard Deviation (Degree)**
0	4.87	4.87	0.9
10	8.79	1.21	1.2
20	15.15	4.85	1.3
30	26.98	3.02	0.8
40	37.00	3.00	0.7
50	52.06	2.06	0.4
60	63.87	3.87	1.1
70	72.35	2.35	0.5
80	81.41	1.41	0.9
90	86.92	3.08	0.8
